# The predictive value of the leuko-glycemic index in spontaneous intracerebral hemorrhage patients undergoing neurosurgical operation: a retrospective cohort study

**DOI:** 10.3389/fneur.2026.1887070

**Published:** 2026-09-03

**Authors:** Jian Sun, Yunhan Zhan, Aiwen Zhang, Mingchao Fan

**Affiliations:** 1 Department of Neurosurgical Intensive Care Unit, The Affiliated Hospital of Qingdao University, Qingdao, China; 2 Department of Central Sterile Supply, The Affiliated Hospital of Qingdao University, Qingdao, China

**Keywords:** Glasgow coma scale, leuko-glycemic index, modified Rankin scale, prognosis, spontaneous intracerebral hemorrhage

## Abstract

**Objective:**

This research sought to determine the prognostic utility of the leuko-glycemic index (LGI) in predicting surgical outcomes following spontaneous intracerebral hemorrhage (ICH), and to establish its potential role in clinical outcome assessment.

**Methods:**

A retrospective cohort study was performed using medical records of spontaneous ICH patients who received surgical intervention at Qingdao University Affiliated Hospital between January 2013 and December 2023. Patients were stratified into two cohorts according to their 90-day modified Rankin Scale (mRS) scores. Associations between inflammatory biomarkers and postoperative prognosis were examined, and a predictive framework was constructed. The discriminatory power and optimal threshold of LGI were determined via receiver operating characteristic (ROC) analysis.

**Results:**

Of the 720 enrolled postoperative ICH patients, 224 (31.11%) achieved a favorable prognosis, while 496 (68.89%) had an unfavorable outcome. Univariate analysis revealed statistically significant disparities in several variables: age, presence of intraventricular hemorrhage, GCS score at admission, timing of surgical intervention, LGI, serum potassium, blood glucose, and heart rate. Binary logistic regression identified LGI (OR, 1.009; 95% CI, 1.004–1.013; *p* < 0.001) as an independent predictor of unfavorable prognosis after ICH surgery. An LGI exceeding 98.38 is associated with a higher likelihood of poor functional recovery, whereas a GCS score of 9 or more tends to predict a more favorable outcome.

**Conclusion:**

Both preoperative LGI and admission GCS score serve as reliable indicators of clinical prognosis in spontaneous ICH patients undergoing neurosurgical operation.

## Introduction

ICH represents one of the most severe and debilitating forms of stroke, accounting for approximately 10% to 20% of all stroke cases (1). Despite advances in clinical management including the adoption of an integrated approach combining pharmacotherapy, surgical intervention, and multidisciplinary care, the overall prognosis of ICH remains unfavorable. More than 60% of affected patients experience mortality or significant disability within 3 months after onset (2).

The postoperative outcome of ICH is determined by a range of factors, such as hemorrhage location, hematoma volume, preexisting comorbidities, and postoperative complications (3). Inflammation plays a central role in the pathophysiology of ICH, with the intensity of the inflammatory response being closely linked to clinical prognosis (4). Within days after ICH onset, white blood cells infiltrate the hematoma site, initiating an inflammatory cascade in the surrounding brain tissue that aggravates blood–brain barrier disruption and exacerbates immune dysregulation. Meanwhile, whether attributable to stress-induced hyperglycemia or underlying diabetes, abnormal glucose level interact synergistically with the inflammatory milieu (5). Hyperglycemia contributes to vascular endothelial injury by enhancing anaerobic glycolysis and stimulating reactive oxygen species generation. Previous studies have demonstrated that both lower admission glucose levels and reduced total leukocyte counts correlate with improved outcomes in patients with acute hemorrhagic stroke (6). In addition, peripheral blood monocytes and lymphocytes have been implicated in the progression of cerebral edema and the induction of neuronal death (7, 8). However, the predictive capacity of individual inflammatory or metabolic markers (such as isolated leukocyte count or glucose level) remains limited. For example, preoperative pulmonary infection may elevate leukocyte counts, complicating the interpretation of prognostic biomarkers (6). In light of these limitations, integrated biomarkers derived from peripheral blood parameters have gained increasing attention in recent research.

The LGI is a composite biomarker that reflects both inflammatory activity and metabolic state. Previous studies have also demonstrated its excellent prognostic predictive utility in diseases such as ketoacidosis, acute myocardial infarction, and the severity of COVID-19 (9–11). Nevertheless, the role of LGI in predicting recovery after ICH has not been thoroughly investigated. Following surgical evacuation of spontaneous ICH, secondary brain injury is largely driven by a toxic interplay between leukocyte-mediated neuroinflammation and stress-induced hyperglycemia. Concurrently, intraventricular extension of hemorrhage often precipitates secondary acute hydrocephalus or ventricular clearance impairment, necessitating tailored management such as external ventricular drainage (EVD) in selected severe or mild cases (2, 12). While isolated blood glucose or white blood cell counts can fluctuate due to transient perioperative factors, the Leuko-Glycemic Index (LGI) combines these parallel pathological cascades. Integrating LGI provides a more stable, biologically comprehensive composite marker for predicting neuroinflammation-induced secondary injury and functional prognosis. The present study employs a large-scale clinical dataset in a retrospective analysis to examine the relationship between LGI levels and postoperative prognosis in patients with spontaneous ICH.

## Materials and methods

### Study population and design

A retrospective review was performed on consecutive patients who underwent surgical management for spontaneous ICH at the Affiliated Hospital of Qingdao University from January 2013 to December 2023. The inclusion criteria comprised: (1) age ≥18 years with a confirmed diagnosis of first-ever spontaneous ICH and subsequent surgical intervention, encompassing either craniotomy or burr hole drainage; (2) availability of all laboratory results obtained within 24 h before surgery. Exclusion criteria were defined as: (1) presence of active or chronic infection; (2) significant pre-existing comorbidities such as malignancy, uremia, hematologic disorders, or autoimmune conditions; (3) documented history of prior cerebral infarction or other significant cerebrovascular disease.

### Data collection

#### General clinical data

The following demographic and clinical variables were recorded: age, sex, body mass index (BMI), smoking status, alcohol use history, and comorbidities including hypertension, diabetes mellitus, and coronary heart disease.

#### Preoperative assessment indicators

GCS score on admission. Systolic blood pressure and heart rate on admission. Radiological findings on head CT or MRI, including the presence of intraventricular hemorrhage and hydrocephalus.

Intraventricular hemorrhage (IVH) was defined as the presence of high-density blood accumulation within any part of the ventricular system on initial non-contrast head CT (13). Beyond simple binary presence, emerging evidence highlights that topographical patterns, structural features, and volumetric extension of intraventricular blood are pivotal prognostic imaging biomarkers (1, 14). To account for IVH severity and volume, the modified Graeb Scale (mGS) was applied by two blinded neuroradiologists/neurosurgeons. The mGS score was incorporated into multivariable models as an independent covariate.

#### Laboratory indicators

All laboratory values were determined using blood samples collected within 24 h before the surgery. These included complete blood cell count, blood glucose level, and electrolyte level. The formula for calculating the LGI is: LGI = White blood cell count (WBC, × 10^9^/L) × Blood glucose level (mmol/L) (15).

#### Treatment and prognosis indicators

Surgical method, postoperative secondary bleeding (defined as new bleeding or expansion of the primary hematoma confirmed by postoperative CT), use of artificial airway (tracheal intubation or tracheotomy), postoperative intracranial infection, situation of tracheotomy, length of hospital stay and length of ICU stay.

Prognosis was evaluated using the modified Rankin Scale (mRS) score at 90 days after surgery. An mRS score of 0–2 was defined as a good prognosis, and a score of 3–6 was defined as a poor prognosis (where a score of 6 indicates death).

### Statistical analysis

All statistical analyses were performed using SPSS version 22.0 (IBM Corp., Armonk, NY). Continuous variables with normal distribution are presented as mean ± standard deviation, whereas skewed data are expressed as median with interquartile range. Categorical variables are reported as frequencies and percentages. Between-group comparisons for categorical data were conducted using the *χ*^2^ test, while the Student’s *t*-test or Mann–Whitney U test was applied for continuous variables, as appropriate.

Variables demonstrating statistically significant associations in univariate analyses were incorporated into a multivariate binary logistic regression model to determine independent predictors of unfavorable outcomes. The discriminative ability of potential predictors was evaluated using ROC curve analysis, with optimal cutoff points identified based on the maximum Youden’s index. A two-sided *p*-value below 0.05 was considered statistically significant for all tests.

## Results

### Univariate analysis

A cohort of 720 patients with cerebral hemorrhage who satisfied the inclusion criteria was enrolled in this study. Based on the 90-day mRS score, the patients were categorized into two groups: a poor prognosis group (mRS 3–6), comprising 496 cases (68.89%), and a good prognosis group (mRS 0–2), consisting of 224 cases (31.11%). The overall median age was 56 years (interquartile range [IQR]: 47–65 years), with 494 (68.6%) of the patients being male. The median hospital stay was 15 days (IQR: 10–24 days), and the median ICU stay was 2 days (IQR: 0–10 days). Both ICU and total hospital stays were significantly prolonged in the poor prognosis group compared with the good prognosis group (5 days vs. 1 day, and 17 days vs. 14 days, respectively). There were 52 deaths within 30 days, corresponding to a mortality rate of 7.2%. A range of clinical indicators demonstrated significantly higher values or incidence rates in the poor prognosis group relative to the good prognosis group (all *p* < 0.05).

#### Demographics and comorbidities

Comorbidities including diabetes, hypertension, and coronary heart disease were more frequent in the poor prognosis group, with rates of 8.1% (40/496), 63.3% (314/496), and 8.7% (43/496), respectively. These figures contrasted with those of the good prognosis group: 3.6% (8/224), 54.9% (123/224), and 3.6% (8/224). The poor prognosis group also had a slightly higher average age (56.5 years vs. 56.0 years).

#### Preoperative disease severity

At admission, the incidence of intraventricular hemorrhage (69.8% vs. 46.4%) and hydrocephalus (24.6% vs. 17.4%) was higher in the poor prognosis group than in the good prognosis group. Similarly, the poor prognosis group presented with elevated median systolic blood pressure (158.5 mmHg [IQR, 140.00–180.00] vs. 151.00 mmHg [IQR, 135.25–170.00]) and heart rate. In laboratory parameters, the LGI was significantly higher in the poor prognosis group (96.54 [IQR, 67.41–142.12]) compared to the good prognosis group (74.89 [IQR, 55.21–96.00]). Conversely, the good prognosis group had a slightly higher serum potassium concentration (3.86 mmol/L vs. 3.70 mmol/L). On admission, the poor prognosis group presented with a significantly lower median GCS score of 7 points (IQR 5–9), contrasting with the higher median score of 11 points (IQR 8–13.75) in the good prognosis group (*p* < 0.05). Detailed data are shown in Table 1.

**Table 1:** The demographic and baseline characteristics.

Variable	Total (*n* = 720)	Prognosis		*p* value
		Favorable (*n* = 224)	Unfavorable (*n* = 496)	
Sex (male)	494 (68.6%)	159 (71.0%)	335 (67.5%)	0.357
Age (years)	56 (47, 65)	56 (46, 63)	56.5 (48, 66)	0.015
GCS	8 (5, 11)	11 (8, 13.75)	7 (5, 9)	<0.001
Hydrocephalus (%)	161 (22.4%)	39 (17.4%)	122 (24.6%)	0.032
Intraventricular Hemorrhage	450 (62.5%)	104 (46.4%)	346 (69.8%)	<0.001
Craniotomy surgery	422 (58.6%)	119 (53.1%)	303 (61.1%)	0.045
Emergency Surgery	556 (77.2%)	141 (62.9%)	415 (83.7%)	<0.001
WBCs (× 10^9^/L)	11.03 (8.31, 14.27)	10.20 (8.10, 12.82)	11.68 (8.45, 14.93)	<0.001
RBC(× 10^9^/L)	4.77 (4.41, 5.12)	4.0.84 (4.44, 5.12)	4.75 (4.38, 5.12)	0.122
Lymphocyte (× 10^9^/L)	1.25 (0.84, 2.08)	1.22 (0.86, 1.74)	1.26 (0.84, 2.19)	0.232
Hemoglobin (g/L)	147.00 (135.00, 158.00)	148.00 (137.00, 158.00)	146.00 (134.00, 157.00)	0.246
Platelet (× 10^9^/L)	205.00 (171.00, 247.00)	198.00 (170.00, 247.75)	210.00 (172.00, 246.00)	0.398
LGI	85.75 (60.13, 123.53)	74.88 (55.20, 96.00)	95.62 (64.45, 139.96)	<0.001
K^+^(mmol/L)	3.77 (3.42, 4.03)	3.86 (3.60, 4.09)	3.70 (3.39, 4.00)	<0.001
Na^+^(mmol/L)	140.00 (137.20, 142.18)	140.20 (137.00, 142.10)	140.00 (137.20, 142.17)	0.592
Cr (μmol/L)	60.83 (49.80, 70.00)	61 (50.90, 70.00)	60.45 (49.53, 73.83)	0.926
UN	5.30 (4.30, 6.60)	5.00 (4.20, 6.15)	5.50 (4.38, 6.75)	0.005
Serum glucose (mmol/L)	7.75 (6.63, 9.56)	7.03 (6.20, 8.16)	8.17 (6.83, 10.03)	<0.001
BMI (kg/m^2^)	25.09 (22.86, 27.55)	24.94 (22.86, 27.98)	25.26 (22.86, 27.34)	0.642
History of hypertension (%)	437 (60.7%)	123 (54.9%)	314 (63.3%)	0.033
History of diabetes (%)	48 (6.7%)	8 (3.6%)	40 (8.1%)	0.025
Smoking (%)	173 (24%)	62 (27.7%)	111 (22.4%)	0.123
Drinking (%)	175 (24.3%)	59 (26.3%)	116(23.4%)	0.393
History of cardiopathy (%)	51 (7.1%)	8 (3.6%)	43 (8.7%)	0.014
Systolic pressure (mmHg)	155.50 (139.00, 176.00)	151.00 (135.25, 170.00)	158.50 (140.00, 180.00)	0.035
Diastolic pressure (mmHg)	90.00 (80.00, 101.00)	90.00 (80.00，100.00)	90.00 (79.25, 102.00)	0.844
HR(bpm)	80.00 (72.00, 92.00)	78.00 (71.25，88.00)	80.00 (72.00, 94.00)	0.003
T(°C)	36.80 (36.50, 37.30)	36.80 (36.50，37.300)	36.80 (36.50, 37.30)	0.862

GCS, Glasgow coma scale; WBC, white blood cells; RBC, red blood cells; LGI, leuko-glycemic index; K+, Blood potassium; Na+, Blood sodium; Cr, Serum creatinine; UN, Urea nitrogen; BMI, body mass index; HR, Heart rate; T, Body temperature.

#### Treatment-related indicators

Elevated rates of artificial airway use (67.1%), tracheotomy (39.3%), and pulmonary infection (60.7%) were observed in the poor prognosis group, contrasting with corresponding rates of 15.6, 3.6, and 27.2% in the good prognosis group. A total of 422 patients (58.6%) underwent craniotomy, with 77.2% of these performed as emergency procedures. Although the craniotomy rate was similar between groups (61.1% vs. 53.1%), the proportion of emergency surgeries was significantly higher in the poor prognosis group (83.7% vs. 62.9%). This group also experienced more postoperative complications, including cerebral infarction (8.1% vs. 1.8%) and cerebral hemorrhage (5.4% vs. 0.4%), alongside a greater need for unplanned re-operation. Detailed data are shown in Table 2.

**Table 2:** Postoperative clinical data.

Variable	Total (*n* = 720)	Prognosis		*p* value
		Favorable (*n* = 224)	Unfavorable (*n* = 496)	
Secondary cerebral infarction (%)	44 (6.1%)	4 (1.8%)	40 (8.1%)	0.001
Secondary hemorrhage (%)	44 (6.1%)	4 (1.8%)	40 (8.1%)	0.001
Rebleeding surgery (%)	28 (3.9%)	1 (0.4%)	27 (5.4%)	0.001
Artificial airway (%)	368 (51.1%)	35 (15.6%)	333 (67.1%)	<0.001
Tracheotomy (%)	203 (28.2%)	8 (3.6%)	195 (39.3%)	<0.001
Length of hospital stay (days)	15 (10, 24)	14 (10, 17)	17 (10, 27)	<0.001
Length of ICU stay (days)	2.00 (0.00, 10.00)	1.00 (0.00, 2.00)	5.00 (0.00, 14.00)	<0.001
Intracranial infection (%)	79 (11%)	17 (7.6%)	62 (12.5%)	0.051
Pulmonary infection (%)	362 (50.3%)	61 (27.2%)	301 (60.7%)	<0.001

ICU, intensive care unit.

### Logistic regression analysis of prognostic factors

Univariate logistic regression analysis revealed a total of 12 factors significantly associated with 90-day prognosis. These were: advanced age (OR, 1.018; 95% CI, 1.006–1.031; *p* = 0.004); lower GCS score (OR, 0.749; 95% CI, 0.710–0.791; *p* < 0.001); presence of hydrocephalus (OR, 1.547; 95% CI, 1.036–2.311; *p* = 0.033) or intraventricular hemorrhage (OR, 2.652; 95% CI, 1.923–3.684; *p* < 0.001); performance of craniotomy (OR, 1.385; 95% CI, 1.007–1.905; *p* = 0.045) or emergency surgery (OR, 3.016; 95% CI, 2.102–4.326; *p* < 0.001); elevated LGI (OR, 1.013; 95% CI, 1.009–1.019; *p* < 0.001); higher admission heart rate (OR, 1.014; 95% CI, 1.004–1.024; *p* = 0.004) and systolic blood pressure (OR, 1.006; 95% CI, 1.001–1.011; *p* = 0.028); and a history of hypertension (OR, 1.417; 95% CI, 1.028–1.952; *p* = 0.033), diabetes (OR 2.368, 95% CI, 1.090–5.147; *p* = 0.029), or coronary heart disease (OR 2.563, 95% CI, 1.184–5.546; *p* = 0.017). The multivariate analysis revealed that both the LGI and the admission GCS score emerged as independent predictors of postoperative outcomes in spontaneous intracerebral hemorrhage patients (*p* < 0.05).

An elevated preoperative LGI level was predictive of a poorer prognosis (OR, 1.009; 95% CI: 1.004–1.013; *p* < 0.001). Conversely, a higher admission GCS score was a protective factor, correlated with more favorable outcomes (OR, 0.794; 95% CI, 0.746–0.844; *p* < 0.001). The complete results of the multivariate analysis are provided in Table 3.

**Table 3:** Univariate and multivariate regression analysis of factors related to prognosis.

Variable	Univariate analysis		Multivariate analysis	
	OR (95% CI)	*p* value	OR (95% CI)	*p* value
Age (years)	1.018 (1.006–1.031)	0.004	1.014 (0.999–1.029)	0.071
GCS	0.749 (0.710–0.791)	<0.001	0.794 (0.746–0.844)	<0.001
Hydrocephalus (%)	1.547 (1.036–2.311)	0.033	0.988 (0.612–1.596)	0.961
Intraventricular Hemorrhage (%)	2.652 (1.923–3.684)	<0.001	0.764 (0.516–1.130)	0.178
Craniotomy surgery (%)	1.385 (1.007–1.905)	0.045	0.846 (0.578–1.237)	0.388
Emergency surgery (%)	3.016 (2.102–4.326)	<0.001	0.945 (0.605–1.477)	0.805
LGI	1.013 (1.009–1.019)	<0.001	1.009 (1.004–1.013)	<0.001
HR (bpm)	1.014 (1.004–1.024)	0.004	1.005 (0.994–1.017)	0.362
Systolic pressure (mmHg)	1.006 (1.001–1.012)	0.028	1.002 (0.996–1.009)	0.486
History of cardiopathy (%)	2.563 (1.184–5.546)	0.017	0.608 (0.258–1.434)	0.256
History of diabetes (%)	2.368 (1.090–5.147)	0.029	0.686 (0.283–1.661)	0.403
History of hypertension (%)	1.417 (1.028–1.952)	0.033	0.887 (0.606–1.297)	0.535
K^+^(mmol/L)	0.560 (0.400–0.782)	0.001	0.862 (0.582–1.275)	0.457

GCS, Glasgow coma scale; LGI, leuko-glycemic index; HR, Heart rate; K+, Blood potassium.

### Predictive value of LGI and GCS score

**Figure 1:**
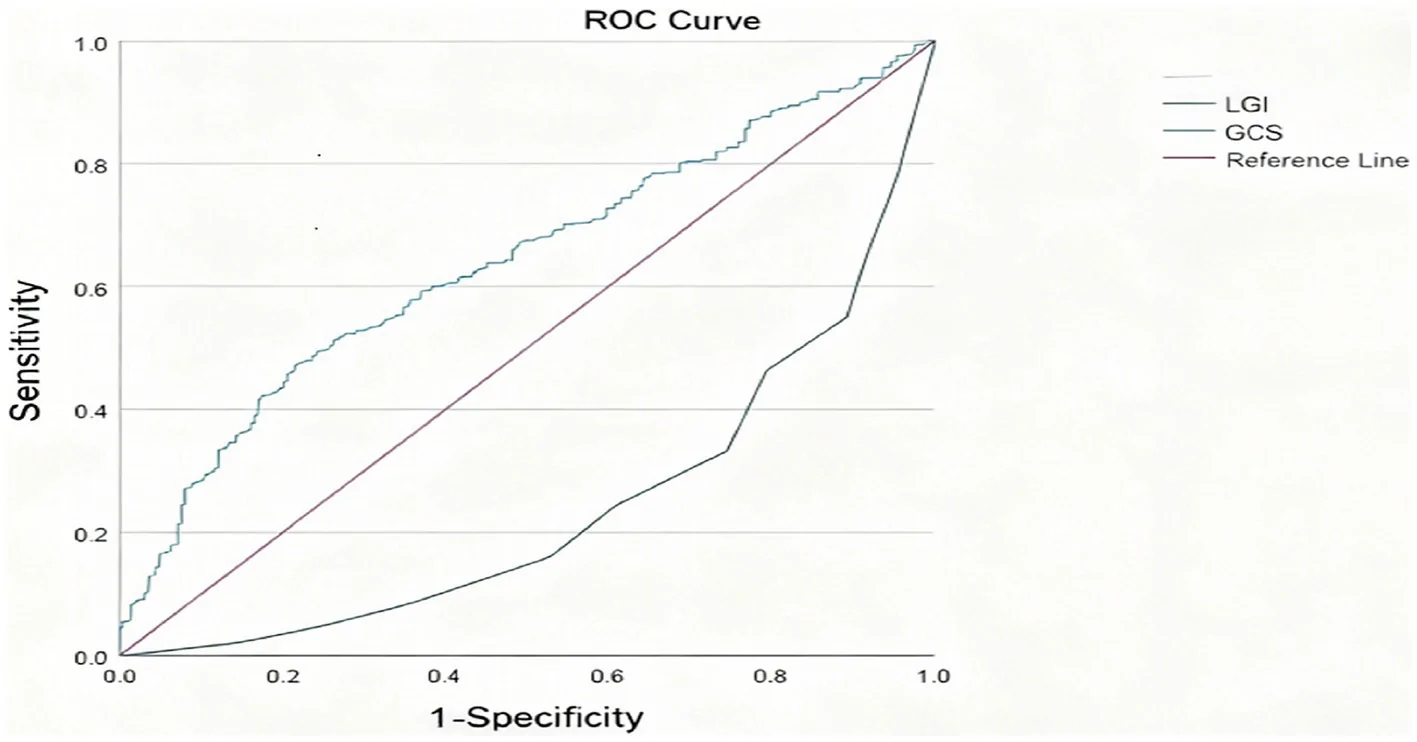
ROC curve of LGI and GCS predicting 90-day outcomes.

The predictive performance of the two independent risk factors, LGI and GCS, was evaluated using ROC curve analysis. Results indicated an optimal LGI cutoff value of 98.38, which corresponded to a sensitivity of 48.4% and a specificity of 78.6% in predicting outcomes after ICH. For the GCS score, the optimal threshold was 8.5, demonstrating a sensitivity of 74.6% and a specificity of 66.7%. Complete ROC statistics, including area under the curve (AUC) values, are summarized in Figure 1 and Table 4.

**Table 4:** Predictive value of SIRI and GCS.

Variable	AUC	*p* value	Cutoff value	Sensitivity	Specificity
LGI	0.662	<0.001	98.377	0.484	0.786
GCS	0.760	<0.001	8.5	0.746	0.667

GCS, Glasgow coma scale; LGI, leuko-glycemic index.

## Discussion

In this retrospective cohort study, we demonstrated that the LGI, a composite biomarker derived from admission leukocyte count and blood glucose levels, serves as a significant predictor of poor prognosis in surgically treated patients with spontaneous intracerebral hemorrhage. An elevated LGI was associated with worse clinical outcomes, with an optimal cutoff value of > 98.38 offering reasonable specificity for prognostic prediction. These findings further corroborate the pathophysiological mechanism underlying the interaction between inflammatory responses and metabolic dysregulation following ICH (16).

The post-hemorrhagic inflammatory cascade is a key driver of secondary injury. White blood cells, particularly neutrophils and monocytes, rapidly infiltrate the peri-hematomal tissue, releasing pro-inflammatory cytokines such as interleukin-6 (IL-6) and tumor necrosis factor-α (TNF-α). This process contributes to blood–brain barrier disruption, exacerbates cerebral edema, and promotes neuronal apoptosis (17, 18). Concurrently, hyperglycemia from either pre-existing diabetes or the acute stress response to severe brain injury can potentiate this damage via multiple mechanisms, including the promotion of anaerobic glycolysis leading to intracellular acidosis, increased generation of reactive oxygen species, and impaired vascular endothelial repair (19). While individual markers like admission leukocytosis or hyperglycemia have been linked to poorer outcomes in ICH (20, 21), their predictive power can be confounded by non-cerebral factors, such as concurrent infections. The LGI, by integrating these two pathways, may offer a more robust reflection of the overall systemic inflammatory-metabolic burden, potentially yielding better prognostic stability than either component alone.

Our study is based on the conclusions of previous research, namely that LGI can serve as an important prognostic indicator for other critical illnesses such as acute coronary syndrome (18, 22) and subarachnoid hemorrhage (23). The present study extends the potential utility of LGI to the realm of spontaneous ICH, a area where its application has been less explored. Of course, our results show that the AUC of LGI in predicting poor prognosis is only 0.662, and its discriminative ability is average. Like most indicators, LGI is more suitable as an auxiliary reference tool rather than an independent decision-making tool.

Beyond LGI, our multivariate model confirmed the well-established prognostic importance of the admission GCS score, a direct indicator of the initial severity of neurological insult. Furthermore, compared with the group of patients with a favorable prognosis, the patients in the group with a poor prognosis had a higher incidence of postoperative rebleeding, secondary cerebral infarction, rebleeding requiring reoperation, need for artificial airways, tracheotomy, and pulmonary complications. This further confirms that clinical complications and the severity of the disease have a significant impact on long-term functional recovery. Such patients usually have more severe initial injuries and are more prone to complications. These factors collectively lead to poorer prognosis, prolonged hospital stay, and increased time spent in the intensive care unit.

Several limitations in our study warrant consideration. First, the LGI was calculated solely based on laboratory values obtained at admission; dynamic changes in LGI during hospitalization were not analyzed, which might have provided additional prognostic information. Second, although we adjusted for multiple confounding factors in the regression model, residual confounding due to unmeasured variables cannot be fully excluded. Future prospective, multi-center studies incorporating serial LGI measurements are needed to validate our findings and further clarify the role of LGI in risk stratification and clinical decision that making for ICH patients. Thirdly, this study was conducted using a single-center retrospective design. Although our sample size is substantial (*n* = 720), the findings may be influenced by center-specific factors, such as institutional patient selection preferences, surgical techniques, and perioperative intensive care management. Consequently, the generalizability of our LGI threshold and findings to diverse healthcare settings remains to be confirmed. Prospective, multi-center, and large-sample clinical research data are needed to verify the universality of the results. Finally, intraventricular hemorrhage (IVH) was operationalized as a binary covariate (presence/absence). Detailed volumetric grading systems (such as the Graeb or modified Graeb scale) were not applied, which limits our ability to evaluate the specific impact of intraventricular blood volume and spatial distribution.

## Conclusion

Both preoperative LGI and admission GCS score serve as reliable indicators of clinical prognosis in spontaneous ICH patients undergoing neurosurgical operation.

## Data Availability

The original contributions presented in the study are included in the article/supplementary material, further inquiries can be directed to the corresponding author.
